# Correction: Utility of a freehand frameless navigation system in computed tomography-assisted ventral bulla osteotomy for otitis media in calves

**DOI:** 10.1371/journal.pone.0345797

**Published:** 2026-03-24

**Authors:** Takeshi Tsuka, Masamichi Yamashita, Yoshiharu Okamoto, Shunsuke Miyazaki, Jun Ishii, Kitaro Yoshimitsu, Yoshihiro Muragaki

The second author’s name is spelled incorrectly. The correct name is: Masamichi Yamashita. The correct citation is: Tsuka T, Yamashita M, Okamoto Y, Miyazaki S, Ishii J, Yoshimitsu K, et al. (2026) Utility of a freehand frameless navigation system in computed tomography-assisted ventral bulla osteotomy for otitis media in calves. PLoS One 21(2): e0342569. https://doi.org/10.1371/journal.pone.0342569
