# Supplementary material for: Utility of a freehand frameless navigation system in computed tomography-assisted ventral bulla osteotomy for otitis media in calves
Source: PLoS One. 2026 Feb 13;21(2):e0342569. doi: 10.1371/journal.pone.0342569 (PMC12904415; doi:10.1371/journal.pone.0342569)
Supplement: Attachment — Submitted filename: Comments for Manuscript, PONE-D-25-45935.docx [file pone.0342569.s002.docx]

This paper represents the first instance of utilizing the freehand frameless navigation system, commonly used in human clinical practice, for animal treatments. We believe that the revisions outlined below will suffice for publication in PLOS One.

As discussed, the operational difficulty with the freehand frameless navigation system is influenced by the target size and its distance from the skin, which should be presented. From the preoperative CT scans, the following details should be included:

1. List the maximum horizontal diameter of the target bulla, and maximum straight diameter relative to it on horizontal CT, and the maximum diameter of bulla on vertical CT if possible.
2. If available, describe the distance from the skin surface to the inferior wall of the

bulla.

3. Note any diagnoses of middle ear disease, such as acute phase of chronic otitis

media or cholesteatoma otitis media infection, made through visual examination before or during surgery.

4. The presence or absence of skull bone destruction and the extent of inflammation

progression to surrounding tissues significantly impact the postoperative course. Therefore, these factors should be considered in the preoperative CT scan.

5. On page 43, line 641: It is important to note that while dizziness may occur due to the spread of middle ear infection to the inner ear, generalized convulsive seizures are unlikely and should be attributed to a brain event. This section requires further consideration. Even if the surgery is performed without issues, high-pressure cleaning in the presence of a skull base bone defect may provoke such occurrences. In human surgery, pressure irrigation is avoided in the middle ear and mastoid cavities, and only saline is used for sufficient cleaning without cytotoxic liquids.

We believe these revisions will enhance the clarity and thoroughness of the paper, ensuring its readiness for publication.
